# Acesso a serviços de atenção hospitalar no período neonatal: análise de redes de deslocamento entre municípios do Estado do Paraná, Brasil

**DOI:** 10.1590/0102-311XPT244422

**Published:** 2023-06-26

**Authors:** Daniela Martins Silveira, Hellen Geremias dos Santos

**Affiliations:** 1 Instituto Carlos Chagas, Fundação Oswaldo Cruz, Curitiba, Brasil.

**Keywords:** Sistema de Informação Hospitalar, Regionalização da Saúde, Serviços de Saúde Neonatal, Atenção à Saúde, Hospital Information Systems, Regional Health Planning, Infant Health Services, Health Care, Sistemas de Información en Hospital, Regionalización, Servicios de Salud Neonatal, Atención a la Salud

## Abstract

Este trabalho objetivou caracterizar internações de residentes no Paraná, Brasil, ocorridas no período neonatal em município diferente do de residência, entre 2008 e 2019, e descrever redes de deslocamento para o primeiro e o último biênio da série, correspondentes aos períodos anterior e posterior a iniciativas de regionalização dos serviços de saúde no estado. Dados sobre internações de crianças com idade entre 0 e 27 dias foram obtidos por meio do Sistema de Informações Hospitalares do Sistema Único de Saúde (SIH-SUS). Para cada biênio e regional de saúde, foram calculados a proporção de internações ocorridas fora do município de residência, a distância média ponderada pelo fluxo dos deslocamentos, bem como indicadores de saúde e de oferta de serviços. Modelos mistos foram ajustados para avaliar a tendência bianual dos indicadores e para verificar fatores associados à taxa de mortalidade neonatal (TMN). No total, 76.438 internações foram selecionadas, variando de 9.030, em 2008-2009, a 17.076, em 2018-2019. A comparação entre as redes obtidas para 2008-2009 e as existentes em 2018-2019 evidenciou aumento no número de destinos frequentes e na proporção de deslocamentos dentro da mesma regional de saúde. Observou-se tendência decrescente para a distância, para a proporção de nascidos vivos com Apgar no quinto minuto ≤ 7 e para a TMN. Na análise ajustada para a TMN, além do efeito de biênio (-0,64; IC95%: -0,95; -0,28), apenas a proporção de nascidos vivos com idade gestacional inferior a 28 semanas apresentou significância estatística (4,26; IC95%: 1,29; 7,06). A demanda por assistência hospitalar no período neonatal aumentou ao longo do período estudado. As redes de deslocamento sugerem impacto positivo da regionalização, embora o investimento em regiões com potencial para se tornarem polos assistenciais seja necessário.

## Abstract (en)

This study aimed to characterize hospitalizations of residents in Paraná State, Brazil, that occurred during the neonatal period in a municipality different from their place of residence from 2008 to 2019, and to describe displacement networks for the first and last biennium of the series, corresponding to periods before and after initiatives to regionalize health services in the state. Admissions of children aged from 0 to 27 days were obtained from the Hospital Information System of the Brazilian National Unified Health System (SIH-SUS) database. For each biennium and health region, the proportion of admissions that occurred outside the municipality of residence, the weighted average distance traveled, and indicators of health and service provision were calculated. Mixed models were fitted to evaluate the biennial trend of the indicators and to investigate factors associated with the neonatal mortality rate (NMR). In total, 76,438 hospitalizations were selected, ranging from 9,030 in 2008-2009 to 17,076 in 2018-2019. The comparison of the networks obtained for 2008-2009 and 2018-2019 revealed an increase in the number of frequent destinations and in the proportion of displacements within the same health region. A decreasing trend was observed for distance, the proportion of live births with 5-minute Apgar ≤ 7, and for NMR. In the adjusted analysis for NMR, besides the biennial effect (-0.64; 95%CI: -0.95; -0.28), only the proportion of live birth with gestational age < 28 weeks showed statistical significance (4.26; 95%CI: 1.29; 7.06). The demand for neonatal hospital care increased over the study period. The displacement networks suggest a positive impact of regionalization, although investment in regions with the potential to become healthcare centers is necessary.

## Resumen

El presente trabajo tuvo como objetivo caracterizar las hospitalizaciones de residentes en el Paraná, Brasil, ocurridas en el período neonatal en un municipio diferente al de su residencia entre el 2008 y el 2019, y describir las redes de desplazamiento para el primer y último bienio de la serie, correspondientes al período anterior y posterior a iniciativas de regionalización de servicios de salud en el Estado. Las hospitalizaciones de niños con edades entre 0 y 27 días se obtuvieron del Sistema de Información Hospitalaria del Sistema Único de Salud (SIH-SUS) y, para cada bienio y regional de salud, se calculó la proporción de ingresos hospitalarios que ocurrieron fuera del municipio de residencia, la distancia media calculada por el flujo de desplazamientos, así como indicadores de salud y de oferta de servicios. Se ajustaron modelos mixtos para evaluar la tendencia bianual de los indicadores y para verificar factores asociados a la tasa de mortalidad neonatal (TMN). En total, se seleccionaron 76.438 hospitalizaciones, que varían desde 9.030 en 2008-2009 a 17.076 en 2018-2019. La comparación de las redes obtenida para 2008-2009 y 2018-2019 mostró un aumento en el número de destinos frecuentes y en la proporción de desplazamientos dentro de la misma regional de salud. Se observó una tendencia decreciente para la distancia, la proporción de nacidos vivos con Apgar al 5º minuto ≤ 7 y para la TMN. En el análisis ajustado para la TMN, además del efecto de bienio (-0,64; IC95%: -0,95; -0,28), solo la proporción de nacidos vivos con edad gestacional < 28 semanas presentó significativa estadística (4,26; IC95%: 1,29; 7,06). La demanda de asistencia hospitalaria en el período neonatal aumentó a lo largo del período estudiado. Las redes de desplazamiento sugieren un impacto positivo de la regionalización, aunque es necesaria la inversión en regiones con potencial para convertirse en polos asistenciales.

## Introdução

Nas últimas três décadas, o Brasil vivenciou um processo de redução das desigualdades socioeconômicas, universalização do acesso aos serviços de saúde e promoção de políticas públicas estratégicas na área de saúde materno-infantil. Esse cenário implicou mudanças no perfil social e reprodutivo das mulheres e nos fatores de risco para a mortalidade infantil, resultando em declínio de óbitos no período pós-neonatal (entre 28 e 365 dias de vida) ^1^.

Por outro lado, novos desafios ganharam evidência, em especial os relacionados à demanda por assistência a mulheres com gestação de alto risco e a recém-nascidos com condições marcadoras de morbidade neonatal grave, como a prematuridade e a asfixia ao nascer ^2^. Tais demandas têm exigido investimentos em infraestrutura e no treinamento de recursos humanos, bem como a configuração de redes de atenção à saúde para a oferta de serviços especializados ^3^.

Atualmente, a mortalidade neonatal, correspondente ao óbito de nascidos vivos entre 0 e 27 dias de vida, é o principal componente do indicador de mortalidade infantil, apresentando, para o Brasil, taxa média de 9,46 óbitos a cada 1.000 nascidos vivos no período de 2007 a 2017 ^4^. Muito baixo peso ao nascer, malformação congênita, asfixia ao nascer, complicações maternas na gestação e peregrinação em busca de assistência para o parto são algumas das condições determinantes desse desfecho ^5^^,^^6^. Tais fatores evidenciam a importância da expansão e regionalização do acesso a serviços de saúde nas áreas obstétrica e neonatal ^7^^,^^8^^,^^9^, combinadas à utilização adequada de tecnologias e práticas assistenciais, sobretudo no parto e imediatamente após o nascimento, quando o atendimento especializado por profissionais habilitados pode ser decisivo para a sobrevida de recém-nascidos com condições marcadoras de risco de vida ^5^.

Esta pesquisa objetivou caracterizar internações de residentes no Paraná, Brasil, ocorridas no período neonatal em município diferente do de residência, entre 2008 e 2019, bem como indicadores de saúde materno-infantil e de oferta de serviços relacionados à assistência aos recém-nascidos no período neonatal segundo regionais de saúde. Buscou-se, ainda, descrever redes de deslocamento para o primeiro e o último biênio da série, correspondentes aos períodos anterior e posterior a iniciativas de regionalização dos serviços de saúde no estado.

## Métodos

Este estudo ecológico, com perspectiva espaçotemporal, analisou dados de internações de recém-nascidos residentes no Estado do Paraná, ocorridas durante o período neonatal (até o 27º dia de vida) em município diferente do de residência da mãe.

Para o período de 2008 a 2019, as informações foram obtidas por meio do Sistema de Informações Hospitalares do Sistema Único de Saúde (SIH-SUS), do Sistema de Informações sobre Mortalidade (SIM) e do Sistema de Informações sobre Nascidos Vivos (SINASC), disponíveis na Plataforma de Ciência de Dados Aplicada à Saúde da Fundação Oswaldo Cruz (PCDaS/Fiocruz) e no Departamento de Informática do Sistema Único de Saúde (DATASUS). Dados sobre leitos de unidade de terapia intensiva (UTI) neonatal (leitos tipo I, II e III) foram coletados no Cadastro Nacional de Estabelecimentos de Saúde (CNES).

Os dados foram agrupados em biênios, considerando três momentos distintos: pré-implantação da Rede de Atenção à Saúde Materno-Infantil no Paraná (2008-2009 e 2010-2011); implantação do Programa Rede Mãe Paranaense (2012-2013 e 2014-2015), assumido como compromisso do Plano de Governo para a Saúde no quadriênio 2012-2014, após instituição da Rede Cegonha no âmbito do Sistema Único de Saúde (*Portaria nº 1.459/2011*) ^10^; e período pós-implantação, com dados disponíveis para internações, nascidos vivos e óbitos infantis (2016-2017 e 2018-2019).

A partir de informações obtidas no SIH-SUS, cada par distinto entre local de residência (origem) e de ocorrência da internação do recém-nascido no período neonatal (destino) representou um deslocamento. Os deslocamentos foram agrupados segundo regional de saúde e biênio e caracterizados pelas seguintes métricas: (a) proporção de internações ocorridas em município diferente do de residência; (b) proporção de internações ocorridas fora do município de residência, porém na mesma regional de saúde; (c) média da aresta de saída média (*m*_*j*_ ) observada para os municípios-origem (*O*_*i*_ ) localizados em uma mesma regional de saúde, ponderada por seu fluxo de saída (*F*_*i*_ ), conforme expressão apresentada a seguir:

mj=Σnji=1Fi×diΣnji=1Fi (Equação 1)

Tal que, *n*_*j*_ = número de municípios na j-ésima regional de saúde, *j* = 1, 2, …, 22; *F*_*i*_ = fluxo de saída do município-origem *i* (*O*_*i*_ ), *i*= 1, 2, …, *n*_*j*_ ; e *d*_*i*_ : aresta de saída média, correspondente à distância média identificada entre *O*_*i*_ e seus *k* municípios-destino (*D*_*k*_ ), ponderada pela frequência de internações (*f*_*k*_ ) ocorridas em *D*_*k*_ , *k*= 1, 2, …, *K*:

di=Σkk=1fk×dOi,DkΣkk=1fk (Equação 2)

Além dessas métricas, foram também calculados por regional de saúde e biênio: (d) número de leitos de UTI neonatal a cada 1.000 nascidos vivos; e (e) indicadores de saúde materno-infantil - proporção de nascidos vivos com peso < 1.500g, com idade gestacional inferior a 28 semanas ou com Apgar no quinto minuto de vida ≤ 7, proporção de mães com 35 anos ou mais e TMN.

Para o primeiro (2008-2009) e o último biênio (2018-2019) do período estudado, redes de deslocamento foram inferidas utilizando grafos definidos por nós e arestas. Cada nó representa a localização espacial da sede do município e cada aresta indica um deslocamento. Os nós apresentam tamanho proporcional ao grau de entrada, que tem como referência o município de ocorrência da internação (destino) e quantifica o número de municípios atendidos em determinado destino. Além dessa métrica, calculou-se também para comparação dos principais destinos o fluxo de entrada, correspondente ao número de internações ocorridas em municípios diferentes dos de origem dos indivíduos. As arestas, por sua vez, são proporcionais ao fluxo de saída, que tem como referência o município de residência da mãe (origem) e quantifica o número de internações de dada origem ocorridas em determinado destino ^8^. Os nós foram coloridos conforme a macrorregião a que pertence o município-destino, e as arestas de acordo com a macrorregião do município-origem ^8^.

Para os biênios (*A*) 2008-2009 e (*B*) 2018-2019, medidas de variação percentual, [(*B - A*)*/A*]*100, foram calculadas tanto para comparar características dos principais destinos (grau e fluxo de entrada) como para comparar a distância média ponderada, a proporção de internações ocorridas em município diferente do de residência e a de internações ocorridas em municípios da mesma regional de saúde.

Por fim, considerando todos os biênios entre 2008 e 2019, realizou-se análise de regressão linear simples com intercepto aleatório (modelo misto) ^11^ para modelar a tendência bianual de indicadores de saúde e de acesso a serviços, bem como da distância média ponderada e da proporção de internações ocorridas fora do município de residência, considerando as regionais de saúde como unidade observacional. Adicionalmente, para a TMN, uma análise de regressão linear múltipla com intercepto aleatório foi ajustada para avaliar sua associação com a distância média ponderada e com os demais indicadores. Foram obtidos estimativas pontuais e respectivos intervalos de 95% de confiança (IC95%) para os efeitos fixos e para o intercepto aleatório, incluído no modelo para acomodar medidas repetidas da variável resposta, em diferentes biênios, na mesma regional de saúde. O processamento e a análise dos dados foram realizados com auxílio da linguagem de programação R (versão 4.2.1, http://www.r-project.org), e o mapeamento das redes foi feito com o programa Gephi (versão 0.9; https://gephi.org/users/download/). O código em R e os bancos de dados estão disponíveis em: https://github.com/Hellengeremias/RedeNeoPR.

## Resultados

Entre 2008 e 2019, foram selecionadas 76.438 internações ocorridas no período neonatal em município diferente do de residência da mãe. A frequência de internações de residentes no Paraná em outros estados brasileiros representou aproximadamente 1% desse total. Para as internações que ocorreram no Paraná, a análise por biênios revelou um aumento progressivo no número de internações (fluxo de saída), que passou de 9.030, em 2008-2009, para 17.076, em 2018-2019, representando 34,67% e 42,52% do total de internações de crianças com até 27 dias de vida ocorridas no estado.

**Figura 1:**
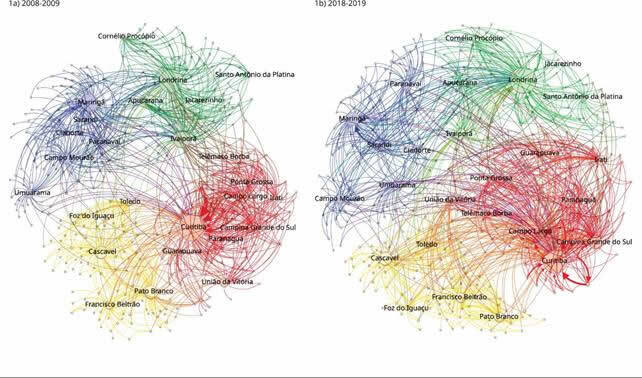
Grafos das redes de deslocamento entre município de residência e de internação no período neonatal. Paraná, Brasil.

Dos deslocamentos que aconteceram no Paraná, observaram-se 2.539 pares origem-destino distintos e distância média, ponderada pelo fluxo de cada deslocamento, de 50,56km. A Figura 1 apresenta grafos de redes de deslocamento para internação no período neonatal em 2008-2009 (Figura 1a) e em 2018-2019 (Figura 1b), correspondentes ao primeiro e ao último biênio do período estudado. O número total de deslocamentos distintos foi de 1.177 e 1.255, com distância média ponderada de 50,35km e 50,71km, respectivamente, em Figura 1a e Figura 1b. Para ambos os períodos, os principais destinos foram representados por municípios-sede das regionais de saúde, com exceção de Campo Largo e Campina Grande do Sul (2ª regional de saúde, macrorregião leste), Santo Antônio da Platina (19ª regional de saúde, macrorregião norte) e Sarandi (15ª regional de saúde, macrorregião noroeste), que apesar de não serem municípios-sede de regional de saúde, se tornaram destinos frequentes no biênio recente, quando comparados a 2008-2009.

Nota: as quatro macrorregiões do Estado estão destacadas em verde (norte), azul (noroeste), amarelo (oeste) e vermelho (leste).

Na Tabela 1 constam o grau e o fluxo de entrada dos principais destinos, destacados nos grafos (Figura 1a e Figura 1b). Alguns destinos apresentaram variação percentual (VP) negativa para o grau de entrada, na comparação dos dois biênios, indicando uma redução no número de municípios atendidos, o que pode ser uma consequência da organização de redes de atenção à saúde materno-infantil. No entanto, todos os destinos, exceto Curitiba e Jacarezinho, exibiram VP positiva para o fluxo de entrada, indicando um aumento na demanda por internações no período neonatal.

**Tabela 1:** Fluxo e grau de entrada dos destinos mais frequentes segundo regional de saúde. Paraná, Brasil, 2008-2009 e 2018-2019.

Macrorregião/Regional de saúde	Município	Grau de entrada			Fluxo de entrada		
		2008/2009	2018/2019	VP	2008/2009	2018/2019	VP
Leste							
1ª	Paranaguá	8	12	50,00	65	218	235,38
2ª	Curitiba	127	108	-14,96	2.740	2.255	-17,70
	Campo Largo (não sede)	48	114	137,50	380	2.571	576,58
	Campina Grande do Sul (não sede)	45	56	24,44	447	638	42,73
3ª	Ponta Grossa	20	26	30,00	61	196	221,31
4ª	Irati	27	39	44,44	215	371	72,56
5ª	Guarapuava	24	24	0,00	171	444	159,65
6ª	União da Vitória	35	39	11,43	184	257	39,67
21ª	Telêmaco Borba	9	7	-22,22	64	97	51,56
Oeste							
7ª	Pato Branco	60	39	-35,00	454	491	8,15
8ª	Francisco Beltrão	32	40	25,00	212	736	247,17
9ª	Foz do Iguaçu	8	11	37,50	100	203	103,00
10ª	Cascavel	54	56	3,70	309	757	144,98
20ª	Toledo	27	23	-14,81	133	308	131,58
Noroeste							
11ª	Campo Mourão	47	34	-27,66	265	707	166,79
12ª	Umuarama	35	40	14,29	212	317	49,53
13ª	Cianorte	11	12	9,09	38	164	331,58
14ª	Paranavaí	49	37	-24,49	221	299	35,29
15ª	Maringá	80	58	-27,50	466	1.305	180,04
	Sarandi (não sede)	4	49	1.125,00	13	273	2.000,00
Norte							
16ª	Apucarana	24	25	4,17	163	482	195,71
17ª	Londrina	111	119	7,21	821	1.251	52,38
18ª	Cornélio Procópio	20	32	60,00	181	392	116,57
19ª	Jacarezinho	6	4	-33,33	7	4	-42,86
	Santo Antônio da Platina (não sede)	13	24	84,62	40	612	1.430,00
22ª	Ivaiporã	80	49	-38,75	356	387	8,71

VP: variação percentual.

A análise dos municípios-origem agrupados por biênio e regional de saúde indica discreta redução na medida de distância entre 2008-2009 e 2018-2019. Das 22 regionais de saúde do estado, 15 apresentaram VP negativa para a distância média ponderada pelo fluxo de saída, com destaque para a 19ª regional de saúde (Jacarezinho, macrorregião norte), que mostrou redução de 123,18km para 59,86km (VP de -51,40). Por outro lado, a 21ª regional de saúde (Telêmaco Borba, macrorregião leste) exibiu aumento de 101,62km para 137,49km (VP de 35,29) entre o primeiro e o último biênio da série estudada. No biênio recente, a 20ª regional de saúde (Toledo, macrorregião oeste) também apresentou medida de distância superior a 100km entre origem-destino (Tabela 2).

**Tabela 2:** Distância média ponderada, proporção de internações ocorridas fora do município de residência e de internações ocorridas em município da mesma regional de saúde. Paraná, Brasil, 2008-2009 e 2018-2019.

Macrorregião/Regional de saúde	Distância média ponderada (km)			Internações [outros municípios] (%)			Internações [outros municípios, mesma regional de saúde] (%)		
	2008/2009	2018/2019	VP	2008/2009	2018/2019	VP	2008/2009	2018/2019	VP
Leste									
1ª	65,32	58,56	-10,36	52,37	54,95	4,93	27,16	54,06	99,04
2ª	23,99	27,26	13,60	31,48	31,40	-0,27	99,63	99,72	0,09
3ª	107,46	94,00	-12,53	26,62	44,38	66,70	15,33	25,33	65,23
4ª	46,68	40,52	-13,20	46,01	69,54	51,12	71,78	87,31	21,64
5ª	78,55	90,45	15,14	30,74	45,92	49,39	75,11	71,63	-4,63
6ª	74,72	63,54	-14,96	41,86	46,25	10,50	72,43	82,24	13,54
21ª	101,63	137,49	35,29	51,50	77,97	51,39	29,61	17,21	-41,88
Oeste									
7ª	45,93	51,82	12,83	38,55	44,91	16,49	93,56	90,75	-3,00
8ª	71,07	65,00	-8,54	44,15	63,62	44,11	78,09	86,23	10,42
9ª	88,08	80,98	-8,07	18,59	23,38	25,79	74,84	87,35	16,72
10ª	79,41	61,84	-22,12	27,46	30,92	12,59	76,92	94,54	22,91
20ª	113,56	101,01	-11,05	32,30	48,13	48,98	53,64	69,34	29,27
Noroeste									
11ª	61,80	55,56	-10,10	49,22	63,55	29,10	76,97	95,51	24,09
12ª	53,19	70,21	31,99	51,51	55,96	8,65	92,68	86,89	-6,25
13ª	71,08	49,78	-29,97	52,60	51,28	-2,52	35,64	62,07	74,16
14ª	67,43	64,02	-5,06	51,86	55,68	7,37	74,06	81,30	9,78
15ª	37,78	28,97	-23,30	34,39	47,72	38,76	85,85	97,31	13,35
Norte									
16ª	41,12	32,48	-21,01	38,63	46,21	19,63	54,70	82,05	50,00
17ª	32,41	31,56	-2,63	25,95	39,85	53,57	96,58	98,50	1,99
18ª	47,02	40,95	-12,92	49,77	71,11	42,88	58,64	86,83	48,07
19ª	123,18	59,86	-51,40	41,37	70,28	69,90	18,93	86,85	358,80
22ª	37,29	47,32	26,91	73,83	75,00	1,59	88,36	81,74	-7,49

VP: variação percentual.

Com relação à proporção de internações ocorridas fora do município de residência, observa-se, em geral, um aumento na comparação entre o primeiro e o último biênio estudado, com destaque para a 21ª regional de saúde (Telêmaco Borba, macrorregião leste), que, além de distância superior a 100km, apresentou, no biênio recente, 77,97% das internações no período neonatal em município diferente do de residência da mãe. Vale destacar que, embora a frequência de internações ocorridas fora do município de residência tenha aumentado, identificou-se maior concentração de deslocamentos dentro da mesma regional de saúde no biênio recente: em 18 das 22 regionais de saúde houve deslocamentos predominantemente intrarregionais (aproximadamente 70% ou mais dos deslocamentos dentro da mesma regional de saúde). Em 2008-2009, 14 regional de saúde apresentaram esse perfil (Tabela 2).

No período recente, ainda há deslocamentos frequentes para outras regionais de saúde. Na macrorregião leste, as regionais de saúde 1ª (Paranaguá), 3ª (Ponta Grossa) e 21ª (Telêmaco Borba) têm como principal destino a 2ª regional de saúde (Metropolitana), com frequências de, respectivamente, 44,7%, 69,8% e 72,8%. Na macrorregião noroeste, destaca-se a 13ª regional de saúde (Cianorte), com frequência de deslocamento para outras regionais de saúde de 38%, predominando como destinos as regionais de saúde 12ª (Umuarama) e 15ª (Maringá), com frequências de 14,2% e 11,8%, respectivamente.

A TMN para o Estado do Paraná variou de 8,98 óbitos/1.000 nascidos vivos, em 2008-2009, a 7,44 óbitos/1.000 nascidos vivos, em 2018-2019. A Tabela 3 mostra a tendência estimada para os indicadores de saúde, de acesso a serviços e de deslocamento, considerando as regionais de saúde como unidade observacional para o ajuste de modelos de regressão linear simples com intercepto aleatório. A TMN apresentou tendência decrescente no período estudado (efeito do biênio de -0,35; IC95%: -0,48; -0,21), bem como distância média ponderada (-0,90; IC95%: -1,79; -0,01) e proporção de nascidos vivos com Apgar no quinto minuto ≤ 7 (-0,06; IC95%: -0,09; -0,03). Os demais indicadores revelam tendência crescente (proporção de internações ocorridas em município diferente do de residência da mãe, de nascidos de mães com 35 anos ou mais, de nascidos vivos com peso < 1.500g e número de leitos de UTI neonatal/1.000 nascidos vivos) ou comportamento constante (proporção de nascidos vivos com idade gestacional inferior a 28 semanas).

**Tabela 3:** Tendência bianual de indicadores de saúde e de características de deslocamento segundo biênio e regional de saúde. Paraná, Brasil, 2008-2019.

Parâmetros estimados	Indicadores de deslocamento		Indicadores de saúde e de acesso a serviços					
	Distância média ponderada (km)	Internações fora do município de residência (%)	Taxa de mortalidade neonatal	Peso ao nascer [< 1.500g] (%)	Idade gestacional [< 28 dias] (%)	Apgar no 5º minuto [≤ 7] (%)	Idade materna [≥ 35 anos] (%)	Número de leitos de UTI neonatal/ 1.000 nascidos vivos
Fixos								
Intercepto	66,29	30,04	9,25	1,14	0,44	2,19	9,85	2,32
IC95%	55,27; 77,31	23,45; 36,64	8,68; 9,82	1,07; 1,21	0,40; 0,47	1,98; 2,39	9,38; 10,31	1,50; 3,14
Biênio	-0,90	6,16	-0,35	0,03	0,01	-0,06	0,88	0,21
IC95%	-1,79; -0,01	5,33; 7,00	-0,48; -0,21	0,01; 0,04	-0,00; 0,02	-0,09; -0,03	0,80; 0,95	0,15; 0,27
Aleatórios								
Intercepto	25,08	14,33	0,94	0,12	0,05	0,43	0,97	1,87
IC95%	18,57; 34,17	10,45; 19,68	0,60; 1,38	0,09; 0,18	0,03; 0,08	0,31; 0,60	0,70; 1,34	1,38; 2,54
Resíduos	8,87	8,35	1,34	0,12	0,08	0,31	0,73	0,62
IC95%	7,78; 10,14	7,33; 9,55	1,18; 1,53	0,11; 0,14	0,07; 0,10	0,27; 0,35	0,64; 0,83	0,55; 0,71

IC95%: intervalo de 95% de confiança; UTI: unidade de terapia intensiva.

A análise de regressão linear múltipla com intercepto aleatório que considerou a TMN como resposta de interesse (Tabela 4) evidenciou ausência de significância estatística para a distância média ponderada (Modelo 2). Na análise ajustada que considerou as demais covariáveis (Modelo 3), além do efeito de biênio (-0,64; IC95%: -0,95; -0,28), apenas a proporção de nascidos vivos com idade gestacional inferior a 28 semanas (4,26; IC95%: 1,28; 7,06) permaneceu associada à TMN.

**Tabela 4:** Tendência bianual da taxa de mortalidade neonatal ajustada para características de deslocamento e demais indicadores de saúde segundo biênio e regional de saúde. Paraná, Brasil, 2008-2019.

Variáveis	Estimativa	IC95%
Modelo 1		
Efeitos fixos		
Intercepto	9,25	8,68; 9,82
Biênio	-0,35	-0,48; -0,21
Efeitos aleatórios (desvio-padrão)		
Intercepto	0,94	0,60; 1,38
Resíduos	1,34	1,18; 1,53
Modelo 2		
Efeitos fixos		
Intercepto	9,20	8,01; 10,43
Biênio	-0,35	-0,48; -0,21
Distância média ponderada (km)	0,00	-0,02; 0,02
Efeitos aleatórios (desvio-padrão)		
Intercepto	0,96	0,58; 1,39
Resíduos	1,34	1,18; 1,54
Modelo 3		
Efeitos fixos		
Intercepto	4,91	0,43; 9,47
Biênio	-0,64	-0,95; -0,28
Distância média ponderada (km)	-0,00	-0,02; 0,02
Internações fora do município de residência (%)	-0,00	-0,05; 0,03
Número de leitos de UTI neonatal/1.000 nascidos vivos	-0,10	-0,35; 0,16
Peso ao nascer [< 1.500g] (%)	0,61	-1,51; 2,45
Idade gestacional [< 28 dias] (%)	4,26	1,28; 7,06
Apgar no 5º minuto [≤ 7] (%)	-0,27	-0,91; 0,44
Idade materna [≥ 35 anos] (%)	0,29	-0,04; 0,59
Efeitos aleatórios (desvio-padrão)		
Intercepto	1,39	0,74; 2,00
Resíduos	1,23	1,05;1,40

IC95%: intervalo de 95% de confiança; UTI: unidade de terapia intensiva.

## Discussão

Observou-se aumento na frequência de internações de crianças com até 27 dias de vida ocorridas fora do município de residência entre 2008 e 2019, porém com maior concentração de deslocamentos dentro da mesma regional de saúde em anos recentes. As redes de deslocamento obtidas para os biênios 2008-2009 e 2018-2019 revelaram que os principais destinos para internação no período neonatal foram representados por municípios-sede das regionais de saúde, embora no biênio mais recente outros municípios tenham ganhado protagonismo como destinos frequentes. A 21ª regional de saúde (Telêmaco Borba) destacou-se tanto pela frequência elevada de internações ocorridas fora do município de residência como pela maior distância entre origem-destino. Embora, individualmente, a TMN e a distância média ponderada tenham apresentado tendência decrescente no período estudado, a análise de regressão linear com intercepto aleatório não indicou associação entre essas variáveis. Apenas a proporção de nascidos vivos com idade gestacional inferior a 28 semanas permaneceu associada à TMN após ajuste para as demais covariáveis.

A partir da década de 1990, o Brasil passou por sucessivas melhorias no campo da saúde pública, impulsionadas pela criação do SUS no fim dos anos 1980, pela implantação do Programa Saúde da Família (PSF), no início dos anos 1990 ^12^ e, mais recentemente, durante a primeira década dos anos 2000, pelo processo de regionalização dos serviços de saúde a partir da estruturação de redes de atenção à saúde.

Tais medidas, em conjunto com políticas de outros setores voltadas à redução de desigualdades socioeconômicas, promoveram mudanças positivas no perfil social e reprodutivo das mulheres, como o aumento da escolaridade, da inserção no mercado de trabalho e do acesso a métodos contraceptivos. Além disso, contribuíram para a redução de fatores de risco para óbitos infantis, sobretudo os ocorridos no período pós-neonatal, com a ampliação do número de consultas durante o pré-natal e o primeiro ano de vida, aumento da cobertura vacinal e melhoria das condições de moradia e de saneamento básico ^13^^,^^14^.

Por outro lado, óbitos ocorridos no período neonatal passaram a representar o principal componente da mortalidade infantil, em especial os ocorridos até o sexto dia de vida ^4^. Para evitar tal ocorrência, são necessários investimentos em infraestrutura e no treinamento de recursos humanos para utilização adequada de tecnologias e práticas assistenciais no parto e imediatamente após o nascimento, visto que marcadores de gravidade no nascimento - como asfixia, prematuridade e baixo peso - e complicações maternas na gestação e durante o parto ^1^^,^^3^^,^^5^ podem levar a esse desfecho.

Tal cenário, por sua estreita relação com a oferta de serviços especializados, requer a configuração de redes de atenção à saúde para organização da assistência hospitalar no período neonatal de modo a reduzir desigualdades de acesso e possibilitar o atendimento em tempo oportuno ^15^^,^^16^. No Brasil, o programa Rede Cegonha ^10^ foi instituído com esse propósito, no ano de 2011, com foco na atenção ao parto, objetivando melhorar a qualidade da assistência ^17^. No Paraná, criou-se, em 2012, a Rede Mãe Paranaense ^18^, uma iniciativa decorrente da experiência exitosa do programa Mãe Curitibana, desenvolvido na capital do estado desde 1999, que tem como objetivo organizar o fluxo assistencial materno-infantil e representou um compromisso do Plano de Governo do Estado para a Saúde no quadriênio 2012-2014.

As redes de atenção à saúde podem ser estudadas mediante a identificação de origens e destinos, correspondentes ao local de residência e de ocorrência do atendimento, retrato que auxilia na avaliação da demanda e na organização da oferta de serviços de saúde de alta complexidade, revelando concentrações e vazios espaciais. Além disso, o conhecimento de características relacionadas ao acesso ao serviço, como disponibilidade de meios de transporte ou distância, tempo e custo envolvidos no deslocamento, também é importante para compreender essas redes ^19^.

Este estudo, ao avaliar a rede de deslocamentos para internações no período neonatal, observou aumento na frequência de internações ocorridas fora do município de residência, provavelmente como consequência da organização do fluxo de saída, em decorrência da rede de atenção instituída e do aumento da demanda por serviços de alta complexidade. Algumas características são descritas como marcadores da necessidade de assistência hospitalar ao recém-nascido, como idade materna ≥ 35 anos, internação da mãe por complicação obstétrica, prematuridade, baixo peso, Apgar no quinto minuto < 7 e malformação congênita ^6^.

Tais marcadores apresentam estreita relação com mudanças no perfil social e reprodutivo das mulheres e no de gravidade das condições de saúde de crianças ao nascer ^6^. Nesse contexto, esta pesquisa verificou que, embora a TMN tenha revelado tendência decrescente no período analisado, a proporção de nascidos vivo com idade gestacional inferior a 28 semanas exibiu associação positiva com esse indicador, reforçando a importância do acesso a serviços de maior complexidade para evitar o óbito neonatal.

No entanto, apesar do aumento de internações fora do município de residência, observaram-se concentração de deslocamentos dentro da mesma regional de saúde e aumento no número de municípios que se destacaram como destinos frequentes, assinalando o impacto positivo da conformação de regiões negociadas e contratualizadas para expansão e regionalização do acesso a serviços de saúde nas áreas obstétrica e neonatal.

Sousa et al. ^8^ analisaram a evolução dos fluxos de deslocamento para a atenção ao parto normal por meio de grafos para o Estado da Bahia e identificaram aumento na proporção de gestantes que realizaram parto normal fora do município de residência, bem como aumento da distância percorrida. Além disso, os autores destacaram o grande fluxo de gestantes para municípios-sede de regiões de saúde como indicador de desigualdades intrarregionais na distribuição de serviços obstétricos.

Neste estudo também foram identificadas regiões que, além do aumento da frequência de internações ocorridas fora do município de residência, apresentam distância média percorrida entre município de residência e de ocorrência da internação superior a 100km, como a 21ª regional de saúde (Telêmaco Borba). Esse dado sugere que a região ainda é carente de serviços especializados ^20^^,^^21^ e que, portanto, pode se beneficiar do planejamento de uma rede de atenção obstétrica e neonatal que assegure à gestante e ao recém-nascido o acesso em tempo oportuno ao serviço com adequado nível de complexidade na definição de referências intermunicipais ^5^.

Nesse sentido, em 2020, um hospital regional estadual foi inaugurado na cidade de Telêmaco Borba, município-sede da 21ª regional de saúde, inicialmente voltado à assistência durante a pandemia de COVID-19. Recentemente, o local passou por uma estruturação de modelo assistencial, visando ofertar serviços especializados na área de saúde materno-infantil, com previsão de leitos de enfermaria, alojamento conjunto para maternidade, berçários e leitos de UTI neonatal ^22^. Esse evento reforça a importância de políticas de regionalização da saúde para identificação de regiões com potencial para se tornarem polos de assistência mediante investimentos estruturais e gerenciais, que superem o predomínio, no cenário atual, de transporte de pacientes entre municípios distantes, condicionado à disponibilidade de vagas de leitos de internação ^9^.

Esta pesquisa apresenta limitações. Os dados são secundários, obtidos de sistemas de informação em saúde, portanto, pode haver erros de registro, subnotificações ou atrasos em seu processamento. Além disso, o banco de dados do SIH-SUS contabiliza somente internações hospitalares financiadas pelo SUS. Por fim, as redes de deslocamento permitem apenas investigar relações intermunicipais do tipo origem-destino determinadas pela presença do serviço e dificuldades relacionadas às possíveis trajetórias, como tempo e custo, não foram analisadas.

O estudo de redes de deslocamento para assistência hospitalar no período neonatal pode auxiliar a gestão regional na organização das referências intermunicipais, de modo a otimizar recursos e serviços e reduzir desigualdades de acesso ^15^^,^^16^. Os resultados desta pesquisa sugerem impacto positivo do processo de expansão e regionalização do acesso a serviços de saúde nas áreas obstétrica e neonatal por meio da conformação de redes de atenção à saúde. Embora tenha sido observado aumento na frequência de internações ocorridas fora do município de residência, houve maior concentração de deslocamentos intrarregionais e aumento da quantidade de municípios que se destacaram como destinos frequentes. Além disso, observou-se tendência decrescente da distância média ponderada pelo fluxo entre 2008 e 2019, ainda que algumas regiões apresentem valor superior a 100km, indicando que há espaço para melhorias na organização das redes regionalizadas no período neonatal, a partir do investimento em regiões com potencial para se tornarem polos assistenciais.
